# Characteristics and Statistical Analysis of Large and above Hazardous Chemical Accidents in China from 2000 to 2020

**DOI:** 10.3390/ijerph192315603

**Published:** 2022-11-24

**Authors:** Dingding Yang, Yu Zheng, Kai Peng, Lidong Pan, Juan Zheng, Baojing Xie, Bohong Wang

**Affiliations:** 1 National & Local Joint Engineering Research Center of Harbor Oil & Gas Storage and Transportation Technology, Zhejiang Provincial Key Laboratory of Petrochemical Pollution Control, School of Petrochemical Engineering & Environment, Zhejiang Ocean University, Zhoushan 316022, China; s20082000015@zjou.edu.cn (L.P.); zhengjuan@zjou.edu.cn (J.Z.); 2 School of Naval Architecture and Maritime, Zhejiang Ocean University, Zhoushan 316022, China; z20086100062@zjou.edu.cn (Y.Z.); pengkai@zjou.edu.cn (K.P.); 3 Key Laboratory of Safety Engineering and Technology Research of Zhejiang Province, Hangzhou 310027, China; xbjing123@aliyun.com

**Keywords:** chemical safety, hazardous chemical, the entropy weight method, TOPSIS

## Abstract

To investigate the occurrence and development pattern of large-scale hazardous chemicals emergencies, a statistical analysis of 195 large and above accidents of hazardous chemicals in China during 2000–2020 was conducted. A general description of the characteristics of larger and above accidents based on statistical data was analyzed, and then the system risk of the hazardous chemical industry was calculated and evaluated by the entropy weight method and the TOPSIS method comprehensively. Results show that: (1) The geographical distribution of large and above hazardous chemical accidents (LAHCA) varies significantly; (2) The high-temperature season has high probabilities of having large and above accidents; (3) Human factors and management factors are the main causes of LAHCA; (4) During the period from 2000 to 2020, due to the rapid development of the chemical industry, the overall risk of accidents involving hazardous chemicals were upswing accompanied by volatility, and the risk of serious accidents remains high. The development history of safety regulations in China’s hazardous chemical sector and the industry’s projected course for future growth were then discussed. Finally, based on the findings of the aforementioned statistics and research, specific recommendations were provided for the safety management of the hazardous chemical sector. This study expects to provide a practical and effective reference for the construction of safety management as well as accident prevention in the hazardous chemical industry.

## 1. Introduction

Advances in science and technology as well as changes in industrial structure are leading to a global increase in demand for diverse petroleum products and chemical feedstocks [1]. According to the American Chemical Abstracts, the annual production of chemicals in the world exceeds 400 million tons [2], and there are 5–7 million kinds of hazardous chemicals known. Hazardous chemicals are prone to leaks, fires, and explosions during production, usage, storage, and transport, which are affected by the properties of raw materials [3]. Safety accidents in the hazardous chemicals industry are an alarming occurrence. For example, the explosion of ammonium nitrate caused by the fire of nitrocellulose cotton in Tianjin port in 2015 [4] and the explosion of ammonium nitrate in the port of Beirut, the capital of Lebanon, in 2020 [5], both caused huge casualties and economic losses. Compared with general occupational safety accidents, the huge scale of accidents in the hazardous chemical industry and the serious danger to public safety make safety management particularly important [6].

Safety management in the hazardous chemical industry has long been a priority in China [7]. Despite the fact that both central and local governments in China have implemented a series of rules, technical standards, and other measures to increase the safety of chemicals. Given the frequency of large incidents, the fact that the accident risk rises with the expansion of chemical industry clusters cannot be disregarded. For example, on 21 March 2019, a series of explosions and fires at a chemical company in Jiangsu, China, killed at least 78 people, injured 640, and caused huge property damage [8]. According to the Department of Supervision and Management of Hazardous Chemicals Safety (DSSMHC) in China, 620 chemical accidents occurred in China from 2016 to 2018, resulting in 728 deaths [9].

Many scholars conducted a qualitative analysis of accident characteristics by statistical accident information. For the risk of hazardous chemical leakage, Hou et al. [10] collected information related to 5207 hazardous chemical leakage accidents and some evacuation caused by hazardous chemical leakage accidents in China from 2009 to 2018 and analyzed the characteristics and development trend of hazardous chemical leakage events and the level of emergency evacuation in China, to construct a comprehensive management framework for hazardous chemicals in China. Zwetsloot et al. [11] studied hazardous chemical incidents in Chester County, Netherlands, and found that chemical spills were more likely to occur in the middle of the day. accidents had a higher probability of occurring at noon on weekdays. Dakkoune et al. [12] analyzed 169 accidents in the French chemical industry and concluded that human factors were the main cause of hazardous chemical accidents (HCA). Descriptive statistics on a large amount of accident information allow scholars to recognize the general characteristics of accidents, but the correctness of conclusions is determined by the depth of accident data mining. To systematically dissect accident information, some scholars constructed various quantitative risk assessment models for hazardous chemicals based on statistical results [13]. Wang et al. [14] analyzed the statistical data of tunnel gas accidents in China using the grey correlation method and obtained the ranking of the influence of the main factors of tunnel gas accidents in China. Chen et al. [15] developed a linear programming model for the collaborative emergency response problem of major emergencies in China. The solution and selection of the optimal method of emergency response plan were proposed. Pan et al. [16] proposed a Bayesian network-based computational model for accident risk based on the statistical information of 159 HCA that occurred in China during 2017–2021 to derive the characteristics and root causes of hazardous chemical accident risk in China. A large number of studies mentioned above show that statistical methods can provide accurate and effective means for risk analysis of the HCA [17]. Meanwhile, the overall quantitative risk evaluation of the hazardous chemical industry in China has rarely been reported in recent years [18], so this paper makes a computational analysis of the overall safety risk of the hazardous chemical industry in China based on statistical accident information.

Most of the methodological approaches to accident data statistics are still largely subjective. This paper attempts to use an objective data processing method for accident risk calculations. The Technique for Order Preference by Similarity to an Ideal Solution (TOPSIS) is a method for ranking the relative merits of objects to be evaluated according to their proximity to an idealized objective in a finite number of evaluation objects [19]. The method is widely used in multi-objective decision-making situations. Jiang et al. [20] analyzed the coal mine safety input decision by entropy-TOPSIS; Shi et al. [21] evaluated the fire risk of student apartments by combining WSR and TOPSIS method; Huang et al. [22] evaluated the risk of China’s railroad hazardous materials transportation system by combining entropy-TOPSIS-coupled coordination method. As the conclusions, we can find that little research on the quantitative wind analysis of the risk of larger and above hazardous chemical accidents (LAHCA) in China. This study applies the entropy-TOPSIS-based method to calculate and rank the comprehensive risk index of the hazardous chemical industry based on the statistical data of LAHCA in China from 2000 through 2020.

The purpose of this research is to create a comprehensive picture and quantitative analysis of the system risk of China’s hazardous chemical industry by methodically analyzing the occurrence pattern and risk characteristics of large and above accidents in that industry from 2000 to 2020. The research questions are as follows: (i) the occurrence pattern of accidents in China’s hazardous chemical industry; (ii) the development trend of systemic risk in China’s hazardous chemical industry in recent years; and (iii) the possible directions of future development of safety management construction in China’s hazardous chemical industry. This study can make recommendations for the future safety management of China’s hazardous chemical industry as well as serve as a guide for other nations with a rapidly growing hazardous chemical industry.

## 2. Materials and Methods

### 2.1. Data Sources

Two sources of data on HCA in China were used for this study: the official website of the Ministry of Emergency Management of the People’s Republic of China and the official website of the China Chemical Safety Association (CCSA). The Ministry of Emergency Management of the People’s Republic of China is a government agency established in 2018, which is the most authoritative source of statistical data on various disaster events (HCA) in China. The China Chemical Safety Association (CCSA), established in 2006, is a social group engaged in chemical safety communication in China. The hazardous chemical accident column of CCSA can provide detailed hazardous chemical accident reports. Numerous researchers chose it as a data source in their research work on accident statistical analysis [23]. In this study, data on 195 cases of LAHCA that occurred in China from 2000 to 2020 were collected.

### 2.2. Methods

#### 2.2.1. Statistical Methods of HCA

According to the “Report on production safety accident and regulations of investigation and treatment”, accident levels can be divided into four categories based on casualties and economic losses [24]: ordinary accidents, large accidents, major accidents, and tremendous devastating accidents as shown in Table 1. In this study, the casualty information of HCA causing three or more deaths in China from 2000 to 2020 was collected through the official website of the Ministry of Emergency Management of the People’s Republic of China and the official website of the China Chemical Safety Association. 195 cases of HCA causing three or more deaths in China from 2000 to 2020 were collected, with a total of 1273 deaths and 3400 injuries in 29 provincial-level regions in China.

In this study, the accident data and reports were statistically investigated to analyze the HCA that occurred in China. First, valid information was extracted from the collected accident information, and then the data was processed to develop an accident information database using excel software. The basic information about accidents is classified and extracted. The number of accidents, injuries, and fatalities were taken as the main indicators, and the statistical results were analyzed in detail by using the method of comparative analysis in terms of area, time, industry, cause, classification, etc., using statistics. The statistical results can be used to analyze the development trend and the characteristics of emergencies of HCA in China.

#### 2.2.2. Analysis Method of Hazardous Chemical Accident System Risk

**Figure 1:**
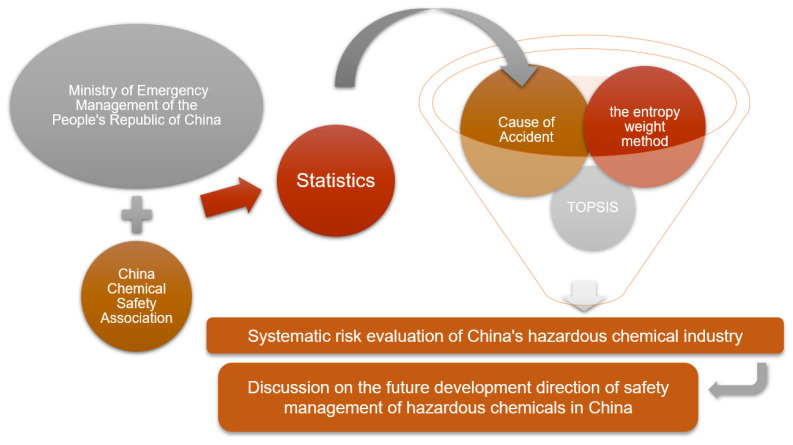
Methodological approach for accident analysis.

First, the causes of accidents are extracted and classified. Four indicators of personnel factor (human), equipment factor (machine), environment factor (environment), and management factor (management) are used as classification criteria and elaborated for each indicator. Then the system risk of monthly (yearly) accident statistics is calculated by using the entropy weight method and TOPSIS, and the calculated results are compared with the statistical results for verification, and the system risk of China’s hazardous chemical industry is evaluated, and finally, improvement suggestions are made according to the evaluation results. The research line of this paper is shown in Figure 1.

#### 2.2.3. Hazardous Chemical Industry System Risk Classification

The causes of HCA are complex and diverse, and the chemical production process requires a series of continuous physical and chemical processing steps from raw materials to products. Therefore, the Work Breakdown Structure (WBS) and Risk Breakdown Structure (RBS) methods can be used to model the possible risks in the production process from the time and system dimensions, respectively [25].

First of all, the WBS decomposition of the hazardous chemical management process is carried out, and the whole hazardous chemical management process is divided into six stages $W_{i}\;\left(i=1,2,3,4,5,6\right)$ : production, storage, use, operation, transportation, and disposal. In addition, each stage contains j (j=1,2,3,…n) sub-processes $W_{i,j}$ according to the time process. Each process can be further divided in detail according to the specific nature of hazardous chemicals. Since the process $W_{i,j}$ needs to satisfy the condition that they do not overlap with each other and have clear boundaries, they can only be subordinated to one of the upper-level units. As shown in Equation (1).

(1) $$W^{T}=\underset{j}{\sum }W_{1,j}\cup \underset{j}{\sum }W_{2,j}\underset{j}{\sum }W_{3,j}\cup \underset{j}{\sum }W_{4,j}\cup \underset{j}{\sum }W_{5,j}\cup \underset{j}{\sum }W_{6,j},\forall ,j$$

where $W^{T}$ denotes the whole process in the hazardous chemical management cycle T .

Then, the RBS decomposition of the hazardous chemical management process is carried out. According to the safety system engineering theory, the risk factors affecting the safety of the hazardous chemical management system can be divided into four major categories $R_{k},\;k=1,2,3,4$ , and each major category can be further divided into l=1,2,3,⋯,m categories of sub-risk factors.

According to the above WBS-RBS division of systematic risk factors in the whole process of hazardous chemical management, all possible risk factors in the whole process can be found. Based on the above results, the risks of hazardous chemical management systems can be identified, and some risk factors are shown in Table 2.

#### 2.2.4. The Entropy-TOPSIS-Based Method

The cause of the accident can be divided into four subsystems: human, machine, environment, and management, divided a fixed period (1 year) into several small periods (by month), the entropy-TOPSIS-based risk evaluation method for hazardous chemical systems counts the frequency of HCA within a period from four perspectives: human, machine, environment and management.

Step 1: Count the number of accidents caused by unsafe factors of human, machine, environment, and management, $u_{i,j}$ , where i = 1, 2, ⋯ , *m*, *m* = 4, denote the four subsystems of human, machine, environment and management respectively; j means the ordinal number of months which should be evaluated, j = 1, 2, …, n,n ∈ N +. From the initial matrix $U={\left[u_{i,j}\right]}_{m\times n}$ :

(2) $$U={\left[u_{i,j}\right]}_{m\times n}=\left[\begin{array}{cccc} U_{1,1} & U_{2,1} & U_{3,1} & U_{4,1}\\ U_{1,2} & U_{2,2} & U_{3,2} & U_{4,2}\\ \vdots & \vdots & \vdots & \vdots \\ U_{1,n} & U_{2,n} & U_{3,n} & U_{4,n} \end{array}\right]$$

Then the probability $t_{i,j}$ of an accident in the j th month for the i th subsystem is:

(3) $$t_{i,j}=u_{i,j}/\underset{j}{\sum }u_{i,j},\forall i,j$$

Step 2: The entropy weight method is used to calculate the weight of the accident occurring in the j th month in the i th subsystem. The smaller the entropy value, the larger the entropy weight, indicating that the more informative the indicator is, the more important the weight of the indicator is. Normalized matrix $R={\left[r_{i,j}\right]}_{m\times n}$ can be formed by Equation (4).

(4) $$r_{i,j}=\frac{{\left(u_{i,j}\right)}_{max}-u_{i,j}}{{\left(u_{i,j}\right)}_{max}-{\left(u_{i,j}\right)}_{min}},\;\forall i,j$$

where: $r_{i,j}$ is the normalized value; ${\left(u_{i,j}\right)}_{max}$ and ${\left(u_{i,j}\right)}_{min}$ are the maximum and minimum values of $u_{i,j}$ respectively.

Calculate the entropy value of the i th subsystem $e_{i}$ :

(5) $$e_{i}=-\frac{1}{\ln n}\sum_{j=1}^{n}f_{i,j}\times \ln\left(f_{i,j}\right)$$

(6) $$f_{i,j}=r_{i,j}/\sum_{j=1}^{n}r_{i,j},\forall i,j$$

When $f_{i,j}=0$ , $f_{i,j}\times \ln f_{i,j}=0$ . Calculate the entropy weight $\tilde{\omega_{i}}$ using the entropy value of the i th subsystem:

(7) $$\tilde{\omega_{i}}=\left[1-e_{i}\right]/m-\sum_{i=1}^{m}e_{i},\;\forall i$$

Step 3: TOPSIS method was used to solve the system risk comprehensive evaluation index $C_{j}$ of the hazardous chemical industry in the j th month (year). The larger its value is, the smaller the system risk is in that month (year) and the safer the system is. This index mainly evaluates the monthly comprehensive risk of hazardous chemical industry in our country from the macro level. Firstly, the weighting matrix $O={\left[o_{i,j}\right]}_{m\times n}$ is calculated:

(8) $$o_{i,j}=\tilde{\omega_{i}}\times r_{i,j},\;\forall i,j$$

Determine the optimal solution $S_{i}^{+}$ and the inferior solution $S_{i}^{-}$ for the i th subsystem weighting value:

(9) $$S_{i}^{+}=\max\left(o_{i,1},o_{i,2},\cdots ,o_{i,n}\right),\;\forall i$$

(10) $$S_{i}^{-}=min\left(o_{i,1},o_{i,2},\cdots ,o_{i,n}\right),\;\forall i$$

The Euclidean distances $d_{i}^{+}$ and $d_{i}^{-}$ of the j th month weight from the optimal and inferior solutions can be calculated by Equations (11) and (12).

(11) $$d_{i}^{+}=\sqrt{\sum_{i=1}^{m}{\left(S_{i}^{+}-o_{i,j}\right)}^{2}},\;\forall j$$

(12) $$d_{i}^{-}=\sqrt{\sum_{i=1}^{m}{\left(S_{i}^{-}-o_{i,j}\right)}^{2}},\;\forall j$$

Calculate the comprehensive evaluation index of hazardous chemical risk $C_{j}$ for the j th month by Equation (13).

(13) $$C_{j}=\frac{d_{i}^{-}}{d_{i}^{+}+d_{i}^{-}},\forall j,C_{j}\in \left[0,1\right]$$

## 3. Characteristics of LAHCA

### 3.1. Provincial Location Distribution

**Figure 2:**
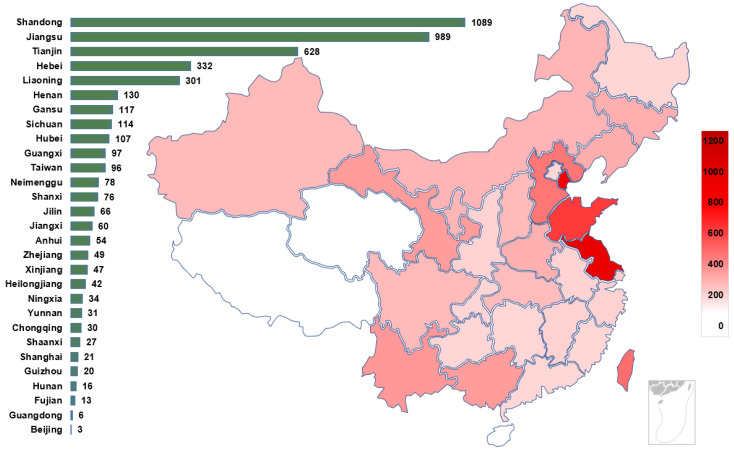
Regional distribution of LAHCA in China. Note: Beijing, Tianjin, Shanghai, and Chongqing are municipalities.

**Figure 3:**
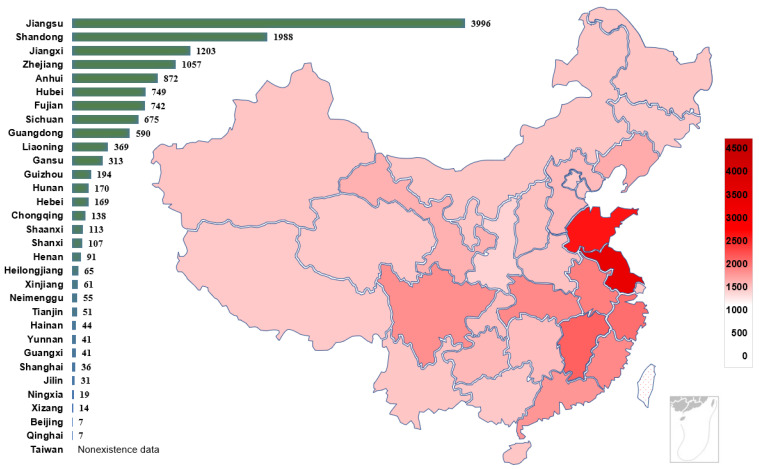
Regional distribution of enterprises in China’s hazardous chemical industry as of 2022 (mainland only). Note: Beijing, Tianjin, Shanghai, and Chongqing are municipalities.

In China, the chemical and petroleum industry is a traditional pillar industry [26]. Therefore, each province attaches great importance to the petrochemical industry and has established a certain number of petrochemical plants. Figure 2 shows the distribution of casualties of these 195 accidents in the provincial areas of China according to the regional distribution of the cases. The darker the color, the higher the number of casualties in HCA. The number of accident fatalities varies greatly by province, with some provinces having far more fatalities than others. Such as Jiangsu Province, with 192 fatalities between 2000 and 2020, accounting for 15.1% of the total fatalities. Figure 3 shows the regional distribution of the number of enterprises in the hazardous chemical industry in China as of 2022. Comparing Figure 2 and Figure 3, it is obvious to see the consistency of the number of hazardous chemical casualties and the regional distribution of hazardous chemical enterprises. The number of enterprises and accident casualties in the eastern coastal provinces of China is much higher than those in other regions. Many studies have noted regional differences in HCA [27], the frequency of accidents was highest in Guangdong Province from 2000–2006, followed by Zhejiang Province and Jiangsu Province. However, the statistical results given in this paper are different from those of the above-mentioned studies. The reason is that many small-scale incidents are not included as statistical subjects. In this study, cases of larger and more serious chemical accidents are chosen and studied. The analysis of these cases is listed in Appendix A.

Additionally, the scale of the firm has an impact on the probability of hazardous chemical incidents. According to Wang et al. [28], the disparity in management and automation levels between small and large-scale businesses makes small businesses more accident-prone. This explains why some areas with fewer hazardous chemical businesses have higher casualty rates. In contrast to places like Jiangsu and Shandong, Tianjin has just 51 hazardous chemical industries as of 2022, as seen in Figure 3. However, Figure 2 shows that Tianjin had the third number of casualties from HCA between 2000 and 2020, behind only Shandong and Jiangsu. This is primarily because there are a lot of large-scale businesses and a lot of dangerous chemicals in the Tianjin area, which increased the size of accidents [29]. For instance, the explosion in Tianjin Binhai New Area in 2015 resulted in 165 fatalities in a single incident.

The 195 accidents were distributed in 29 provinces in China, and the frequency of accidents was characterized as high in the east and low in the west, as shown in Figure 2. This is similar to the temperature distribution in the hot season. Therefore, it is important to consider the impact of high temperatures on the frequency of accidents in addition to the variable levels of the economic development [30]. In general, the large regional disparity is an important feature of HCA in China. Regional differences in industry development affect the frequency of accidents. As coastal provinces such as Jiangsu and Shandong develop more petrochemical industries to maximize their transportation and resource advantages, the risk of larger and higher accidents increases.

### 3.2. Time-Volatility Characteristics

**Figure 4:**
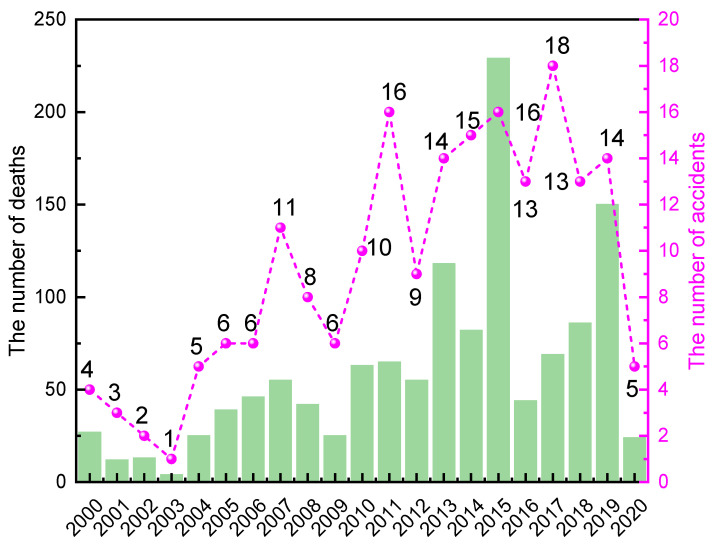
The number of HCA and deaths in different years from 2000 to 2020.

The 195 accidents were classified according to the time of occurrence. Figure 4 shows that the number of accidents involving larger and above hazardous chemicals nationwide in 2003 and 2020 is at a low point, which is directly related to the SARS [31] outbreak in 2003 and the COVID-19 outbreak in 2020. Due to the government’s control of personnel during the epidemic, most enterprises were shut down and the number of accidents dropped significantly. The number of HCA in the country is slowly increasing between 2003 and 2019, which is also the period from the 11th to 13th Five-Year Plan of China’s industrial construction. Due to the rapid development of the chemical industry nationwide, the number of registered hazardous chemical enterprises has also increased significantly, and the frequency of HCA has also increased.

**Figure 5:**
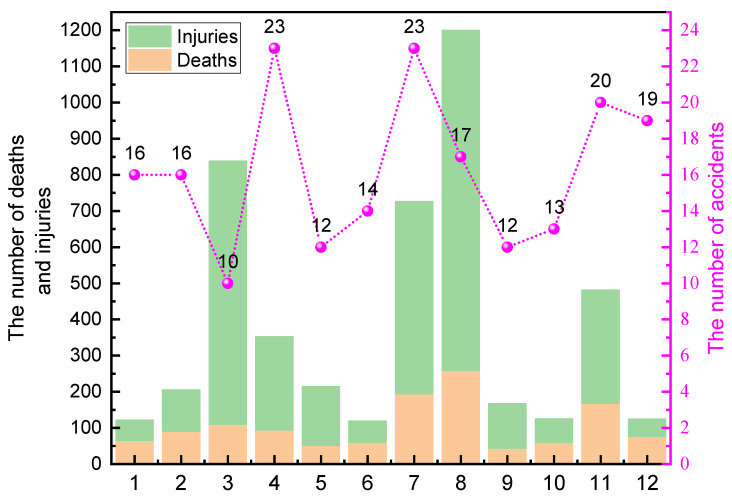
The number of HCA and casualties in different months from 2000 to 2020.

There are large fluctuations in the monthly distribution of HCA in China. The majority of mishaps and fatalities in the first half of the year happened in March and April, as illustrated in Figure 5. The Chinese New Year occurs in January and February, while the majority of Chinese businesses resume operations in March and April. These events bring a significant influx of people and potentially dangerous substances, which frequently results in accidents [32]. The number of accidents and fatalities in July and August in the second half of the year is significantly higher compared to other months, which are two of the hottest months in China [33], and the highest number of casualties in China each year is also in July and August. The release of energy in the event of an accident is directly related to the temperature, and most hazardous chemicals are more likely to diffuse in a high-temperature environment than in a low or ambient environment, making them more likely to cause accidents. High temperatures can have a significant impact on chemical reactions [34]. In their study, Wang et al. [28] examined the features of dangerous chemical mishaps that occurred in China during the high-temperature season. Employees are more susceptible to attention and exhaustion in hot environments, which can lead to dangerous conduct and serious accidents.

### 3.3. Business Type

**Figure 6:**
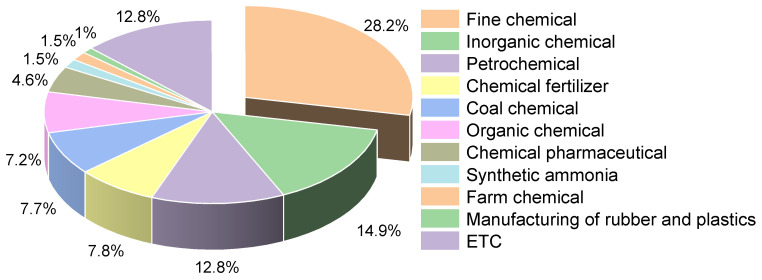
The percentage of HCA and casualties in different industries from 2000 to 2020.

**Figure 7:**
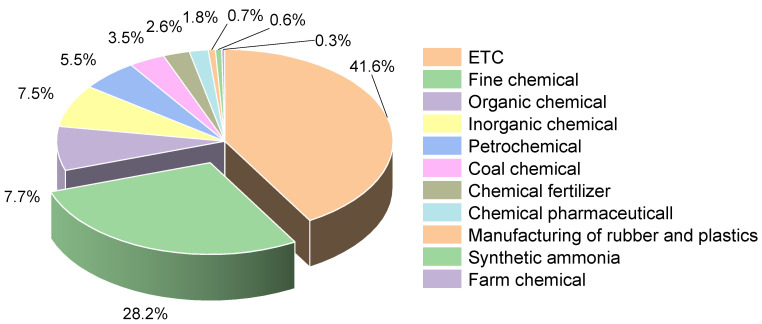
The percentage of casualties in different industries from 2000 to 2020.

Chemical manufacturing encompasses a pretty broad spectrum of industries and a vast range of chemical products. In this study, 195 collected accidents were divided into eleven categories: fine chemicals, inorganic chemicals, organic chemicals, petrochemicals, coal chemicals, chemical fertilizers, chemical pharmaceuticals, rubber and plastic manufacturing, synthetic ammonia, farm chemical, and others. Most of the accidents were concentrated in the fine chemical, inorganic chemical, and petrochemical industries. As shown in Figure 6, where the highest percentage of accidents was in the fine chemical industry, with 55 accidents accounting for 28.2% of the total number of accidents. Figure 7 shows that the number of accident casualties in the fine chemical industry in the same proportion of all industries is higher, the total number of casualties reached 1317, accounting for 28.2% of the total number of casualties, which is directly related to the characteristics of the fine chemical process long, unit reaction, raw materials complex [35], etc. Fine chemical manufacturing processes are characterized by highly variable, challenging-to-control conditions, and a large-scale management system. Additionally, any issue that arises during any stage of this continuous production process will result in a transfer of risk. When emergency measures are not maintained, the situation is frequently serious and challenging to control. For instance, a dichlorobenzene plant explosion happened in a fine chemical plant in Lianyungang in 2017 as a result of chemicals from the tail gas treatment system cascading into the reactor and violently reacting to generate high pressure, which caused an explosion in the workshop when the pressure was released, and the adjacent plant also collapsed by the impact, killing 10 people [36]. China’s fine chemical industry is relatively weak compared to developed countries [37], the safety management level is also relatively backward, and the development process of the fine chemical industry does not match the level of safety, which leads to the frequent occurrence of large-scale accidents.

The number of accidents and fatalities in the synthetic ammonia industry, pesticide industry, rubber and plastic manufacturing industry, and chemical-pharmaceutical industry is at a comparatively low level between 2000 and 2022. These industries fall under the category of hazardous chemical processing businesses and are situated in the middle of the chain of hazardous chemical industries [38]. The majority of these businesses do not produce chemical raw materials. Thus, the production scale and process hazards have been reduced, and the probability of large-scale accidents is also greatly reduced.

### 3.4. Accident Types

The manufacturing of hazardous chemicals involves a complicated process, and the operational setting frequently includes high or low temperatures, high pressures, and corrosion, which increases the likelihood of numerous hazardous chemical incidents. In this paper, HCA are divided into four categories according to accident types: fire, explosion, poisoning choke, and others [39]. The specific distribution and statistical results of the number of casualties in each type of accident are shown in Table 3. Explosion is the most important form of energy release of hazardous chemicals and the most serious type of accident with the most serious consequences, and it is also the most important source of injuries in HCA. The number of accidents with explosions is 115, accounting for 58.97% of the total number of accidents, and the number of casualties caused is 3877, accounting for 82.97% of the total number of casualties. It is worth noting that among all types of accidents, most of the explosions and fire accidents were caused by leaks, with 92 of the 195 larger as well as on accidents directly related to leaks, and 47.15% of the fire and explosion accidents were caused by the evolution of leaks. This trend is consistent with the mechanism of HCA. After a leak occurs, a variety of uncertainties cause accidents to evolve into fires and explosions, and the losses from the consequences of accidents keep increasing.

**Figure 8:**
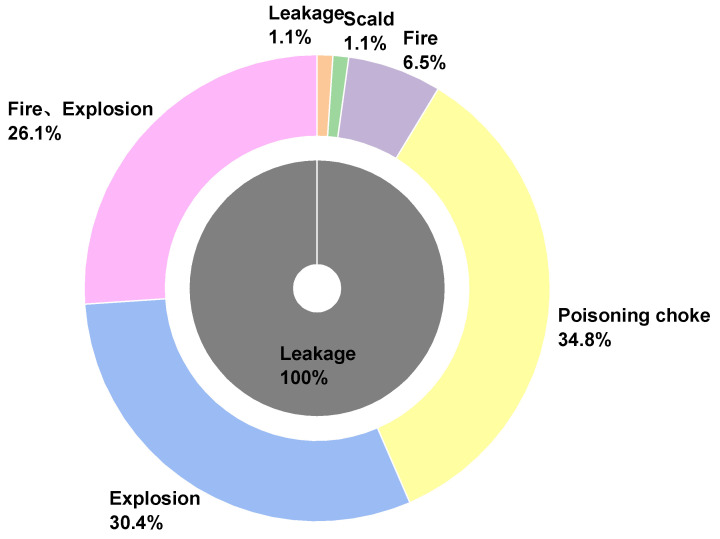
Percentage of accidents caused by leakage.

Leakage is the most common form of failure of the chemical production equipment [40]. Due to the wide variety and nature of hazardous chemicals and different triggering conditions of leaks, the existing preventive measures cannot eliminate the possibility of sudden leakage accidents [41]. And as can be seen from the percentage of spill accident types (Figure 8), there was only one single type of spill accident among 92 spills, and 97.8% of spills resulted in poisoning and asphyxiation, fire, and explosion accidents. Therefore, in the development process of hazardous chemical emergencies, leaks often act as the initiating event of larger accidents and above, which means that leaks are at the front end of the chain of hazardous chemical emergencies. Moreover, the form of uncontrolled release of hazardous chemical energy is also affected by the mechanism of leakage and the nature of hazardous chemicals.

## 4. Risk Evaluation of China’s Hazardous Chemical System from 2000 to 2020

### 4.1. Initial Data Analysis

The statistical periods of month and year were used to analyze the data of safety accidents of hazardous chemicals that occurred in China from 2000 to 2020. The annual and monthly frequencies of the causes of accidents are shown in Table 4 and Table 5.

(1) From 2000 to 2020, the number of accidents caused by human factors related to LAHCA is the highest, reaching 138. The number of accidents caused by environmental factors is the lowest, with only 13 accidents.
(2) From 2000 to 2020, the accidents caused by human, machine, environmental, and management factors of LAHCA are mostly concentrated from April to August.

### 4.2. Evaluation Results

#### 4.2.1. Reliability Analysis

This study stands in the perspective of macro-system risk analysis by collecting historical data and calculating based on the frequency (probability) of occurrence of the corresponding risk factors. The TOPSIS method has some advantages over the N-K model [42] commonly used in multi-factor data analysis:

(1) The N-K model does not consider the impact of time advancement on the hazardous materials management system, and the focus of its examination is on the risk situation in the whole system within a certain period (usually determined by the managers or scholars according to the calculation needs) [43]. Therefore, when collecting historical data, it is only necessary to count the number of incidents that occurred in the system during a certain period;
(2) The entropy-TOPSIS-based model considers the development level of systematic risk in the DG management system during a certain period and focuses on the intensity and development level of subsystems. Therefore, when collecting historical data, it is necessary to count the frequency of certain risk factors in each subsystem in each year (or month);
(3) The entropy-TOPSIS-based model generally adopts the values commonly used in physics when analyzing the system risk intensity, while the N-K model only needs to rank the calculation results [42];
(4) This study focuses on the evaluation of the overall risk of HCA from 2000 to 2020, but the coupling between the causes of HCA is not analyzed, after which modeling studies can be conducted on the intrinsic factors and connections of accidents in the whole process of hazardous chemical management.

#### 4.2.2. Analysis of Calculation Results

**Figure 9:**
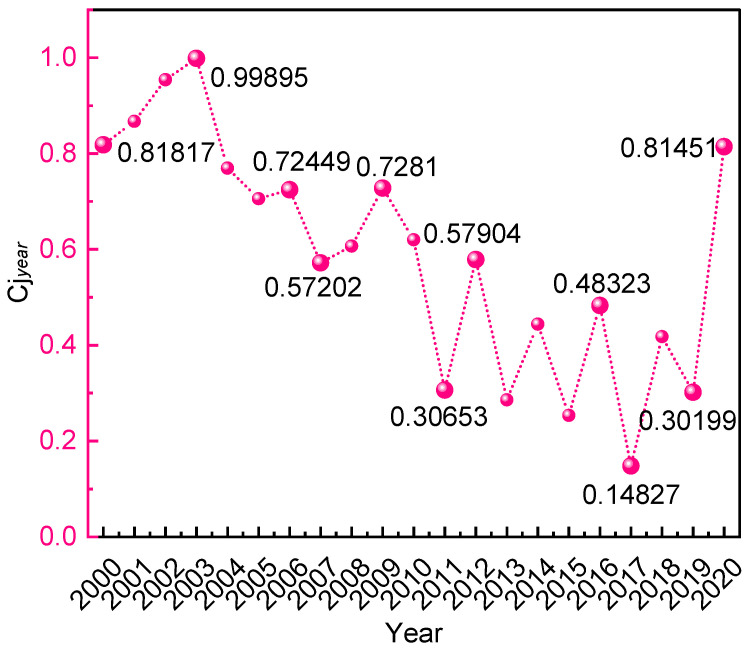
Curve of annual systematic risk factor.

**Figure 10:**
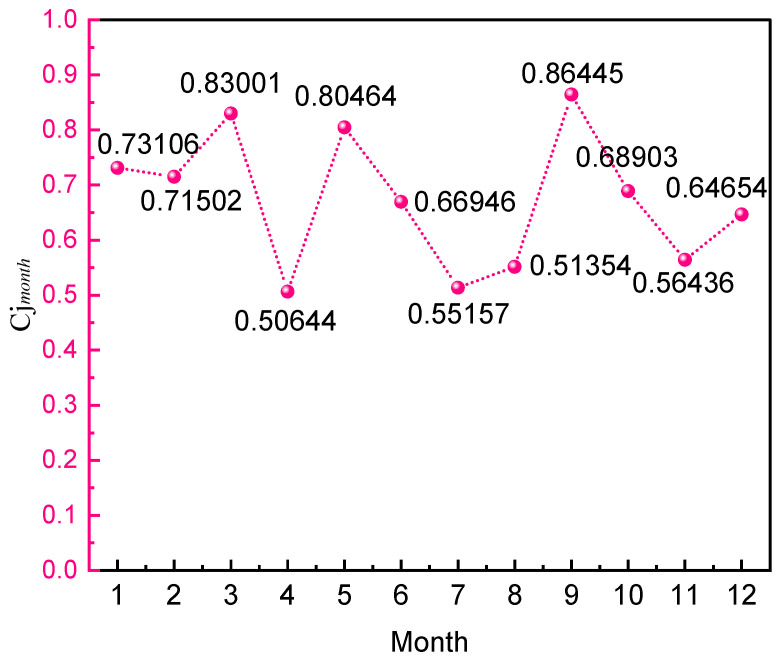
Curve of monthly systematic risk factor.

The comprehensive risk evaluation index $C_{j}$ reflects the magnitude of system safety, the smaller the value, the more dangerous the system is, and vice versa, the safer it is and is used for the longitudinal evaluation of the system. Based on Equation (13), the calculation is carried out for monthly and annual initial data, and the results are shown in Table 6 and Table 7 and Figure 9 and Figure 10, and the calculation results correspond to the statistical results in the previous part.

From the perspective of the yearly calculation results:

- Between 2000 and 2020, China had the highest comprehensive risk index of hazardous chemicals in 2003, which means that the risk of HCA was at the lowest. In 2003, China had an outbreak of SARS, domestic production was stagnant, and the risk of accidents in the hazardous chemical industry was at a low point.
- After 2003, the curve shows a general downward trend, and the system safety fluctuates greatly from 2010 to 2019, indicating that the risk of HCA in China during this period is continuously rising and accompanied by certain fluctuations, which corresponds to the statistical results in the first half of this paper.
- The comprehensive risk index rises and the accident risk decreases in 2020, which is due to the COVID-19 outbreak and the nationwide reduction in production activities, and the risk of hazardous chemical safety accidents decreases.

From the perspective of the monthly calculation results:

- The comprehensive risk index of the first quarter in the annual curve of China’s hazardous chemical safety accident risk from 2000 to 2020 is in a higher position compared with other quarters, which means the hazardous chemical safety risk in the first quarter is at a low point in the whole year. China’s traditional Spring Festival period is in this quarter, and the production activities are slower compared with other months, and the safety requirements of China’s safety supervision departments for production units are higher during the Spring Festival The risk of accidents is at a low level.
- The yearly curve of hazardous chemical safety accident risk in China from 2000 to 2020 is located at the lowest point in April, that is, the safety level of HCA in China is high in April. In March, most enterprises in China just resumed work from the Spring Festival holiday and started to return to production activities on a large scale. A large number of chemical equipment was restarted from hibernation and the risk was elevated accordingly, reaching the highest in April.
- From 2000 to 2020, the risk of hazardous chemical safety accidents in China is at a low point in July and August, indicating that these two months are high-risk times. The two hottest months in China are July and August, which have a direct correlation with the distribution of high-temperature seasons in that country. Under high temperatures, staff members’ physiology and psyche are more prone to laziness and exhaustion, which increases the probability of accidents.

The first phase starts from 2000 to 2010. The national hazardous chemical management system’s risk remained elevated. The level of chemical production, storage, transportation, operation, use, and disposal was raised by the Chinese government through significant financial investment and the implementation of several regulatory rules. The Regulations on the Control over Safety of Dangerous Chemicals promulgated in 2002 and updated in 2011, served as China’s most important legal foundation at the time for the safe management of hazardous chemicals. Due to the weak deterrence, constraint, and disciplinary effects of this rule, the risk of accidents in the hazardous chemical business did not decrease.

The second phase is from 2010–2020. The growth rate of the number of LAHCA in China has slowed down significantly. To some extent, the number of accidents was related to the amount of money invested in emergency management. China was in the implementation phase of the 12th Five-Year Plan (2011–2015) [44] and the 13th Five-Year Plan (2016–2020) [45]. Even if the national strategy encouraged the growth of the chemical sector, serious accidents occasionally happened. For example, the Qingdao oil pipeline explosion caused 62 deaths in 2013, the Tianjin Port “8.12” explosion caused 165 deaths in 2015.

## 5. Discussion

This study looked into and examined 195 larger and greater HCA that occurred between 2000 and 2020 to identify the occurrence pattern of such accidents in China’s hazardous chemical business. The larger and above hazardous chemical mishaps in China were first given a general description before being calculated and verified using the entropy-TOPSIS-based method. Therefore, this section aims to discuss the construction of safety standards in China’s hazardous chemical industry, respond to the questions raised in the introduction, and predict the future course of safety management in China’s hazardous chemical industry based on the available results.

### 5.1. Law Making

**Figure 11:**
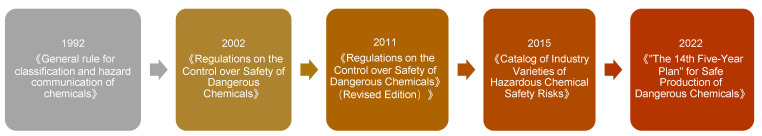
China’s hazardous chemical industry safety standards construction history.

The hazardous chemical industry is one of the important pillar industries of the national economy, and the safety of its production, transportation, and storage process becomes more and more important. The upstream of the hazardous chemical industry is mainly the chemical raw material production industry, including oil and gas extraction and transportation, refining, and chemical product processing and manufacturing process, the midstream is the hazardous chemical production industry, and the downstream industry is the process of hazardous chemical transportation, storage, and distribution. As shown in Figure 11, in 1992, the “General rule for classification and hazard communication of chemicals” was released, marking the completion of the standard construction of China’s classification of dangerous chemicals. 2002, to strengthen the safety management of dangerous chemicals and prevent and reduce dangerous chemical accidents, the State Council issued the “Regulations on the Control over Safety of Dangerous Chemicals”. 2011, the State Council revised the “Regulations on the Control over Safety of Dangerous Chemicals”. In 2015, to deeply learn the lessons from the “8–12” explosion in Tianjin Port, the State Council issued the “Catalog of Industry Varieties of Hazardous Chemical Safety Risks”. 2022, according to the “14th Five-Year Plan”, the State Council issued the “Catalogue of Industries Involving Safety Risks of Hazardous Chemicals”. In 2022, according to the national emergency response system planning and production safety planning, the Ministry of Emergency Management issued the “14th Five-Year Plan” for the production safety of dangerous chemicals, again regulating the safety development of the dangerous chemical industry.

In general, the construction of safety standards for hazardous chemicals in China is developing from rough to meticulous and using the experience and lessons learned from serious and sudden HCA to supplement and improve. From the perspective of EU safety accident legislation, Germany first promulgated the Störfallverordnung in 1980, requiring companies to develop and implement accident prevention measures [46]. On this basis, the EU formulated the Seveso-I-Directive in 1982, which has been continuously improved and revised along with the summary of some typical major accidents [47]. This also shows the importance of the construction of safety standards for hazardous chemicals is gradually being paid attention to in China. A major element of China’s construction of safety for hazardous chemicals is the prevention of large-scale accidents.

### 5.2. Safety Management

**Figure 12:**
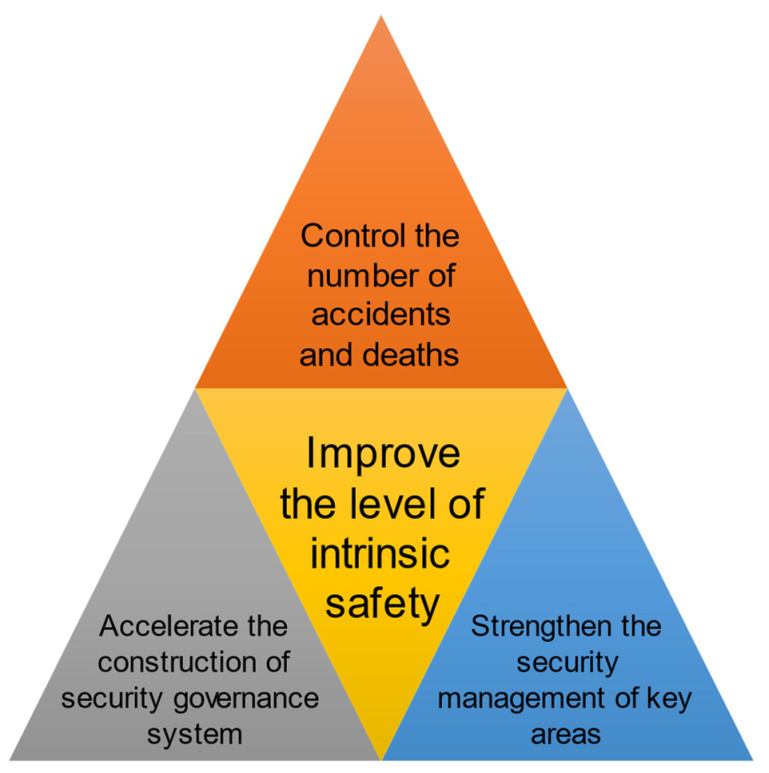
China’s hazardous chemical industry safety management development goals.

On 10 March 2022, China’s Ministry of Emergency Management issued the “14th Five-Year Plan for the Safe Production of Hazardous Chemicals” [48], which specifies the development goals of the hazardous chemical industry for the 14th Five-Year Plan as shown in Figure 12.

With the rise of new industries such as smart logistics and smart parks, leading technologies such as smart warehousing, vehicle-cargo matching, drones, driverless, unmanned terminals, and logistics robots will be more widely used to help the healthy development of the hazardous chemical industry. The modern supply chain is becoming the main driving force for the transportation of hazardous chemicals.

The park has become an important logistics carrier for hazardous chemicals. The development and construction of China’s chemical zone are mostly located in coastal, riverine, chemical economy key areas and chemical resource production areas, which are close to ports and terminals and public and railroad transportation routes, providing convenient conditions for the development of hazardous chemical warehousing logistics; and the abundant resources and high-density petrochemical enterprises also provide hazardous chemical warehousing enterprises with sufficient sources of goods and stable market demand. According to China’s current policy, all new and relocated hazardous chemical production and storage enterprises must enter the professional chemical park, and the chemical park will become the main carrier for the survival and development of hazardous chemical storage enterprises.

## 6. Conclusions

Based on the statistical analysis of 195 LAHCA in China from 2000 to 2020, this paper makes a comprehensive assessment of the safety risks of the hazardous chemical industry using statistical methods from the data of HCA. The main conclusions are as follows.

1. The distribution of large and above accidents in China varies greatly by region, mostly occurring in the eastern coastal areas, which is directly related to the advantages of transportation and resources in coastal areas; HCA are influenced by temperature, and the frequency of accidents in hot months is significantly higher than the rest of months; due to the influence of enterprise scale, chemical raw material production enterprises are more likely to have accidents than hazardous chemical processing enterprises; explosion accidents are explosion is the most important form of injury in LAHCA in China, and leakage of hazardous chemical is the most important trigger of LAHCA.
2. The risk of LAHCA in China from 2000 to 2020 is calculated using the entropy-TOPSIS method. The calculation results can correspond well to the statistical results. The results show that: the number of LAHCA caused by human factors is the highest; the overall risk of LAHCA in China is on the rise after 2003, with serious accidents occurring from time to time, and the turnaround in 2020 is because the COVID-19 epidemic has a greater impact on industrial production.
3. Some particular recommendations are provided for the safety management of China’s hazardous chemical enterprises in light of the characteristics of China’s HCA:
  - Adopt more advanced management techniques and means. For example, check the physical and mental health of staff on time, and focus on the assessment of personnel operation.
  - Improve relevant laws and regulations and safety production rules and regulations, strengthen the preparation of plans and drills, pay attention to safety education and management, and implement safety defense measures, etc.
  - Reduce the probability of problems in all aspects of human, machine, environment and management in the production system of hazardous chemicals, to control the occurrence of HCA.
  - Hazardous chemical companies need to address the unique and complicated nature of safety products in the summer heat, find weak points and unresolved issues, and take effective action to identify and address safety dangers and prevent catastrophic catastrophes.

## Figures and Tables

**Table 1:** Accident levels and definitions of China.

Accident Levels	Definition
ordinary accident	accidents causing less than 3 deaths, or less than 10 serious injuries, or direct economic losses of less than 10 M CNY
large accident	accidents causing more than 3 deaths, or more than 10 serious injuries, or more than 50 serious injuries, or direct economic losses of more than 10 M CNY and less than 50 M CNY
major accident	accidents causing more than 10 deaths, or more than 30 deaths, or more than 50 serious injuries, or direct economic losses of more than 50 M CNY and less than 100 M CNY
tremendous devastating accident	accidents causing more than 30 deaths or more than 100 serious injuries, or direct economic losses of more than 100 M CNY

**Table 2:** List of accident risk factors.

	First Level Indicator Layer	Second Level Indicator Layer	Third Level Indicator Layer
Causes of accidents	Personnel factor (human)	Operators	Not trained for induction
			Violation of rules and regulations
			Improper operation
			Lack of necessary professional knowledge
			Physical or psychological factors
		Rescue workers	Lack of necessary rescue knowledge
			Not wearing their own protective equipment
			Blindly rescuing
	Equipment factor (machine)	Hazardous chemicals	Accumulation of hazardous chemicals
			Mixing of different chemicals
		Production equipment	Inadequate maintenance
			Illegal modification
			Lack of safety protection facilities
			Defective equipment
		Safety and protective equipment	Failure of rescue equipment
			Insufficient quantity
	Environmental factors (environment)	Indoor and outdoor temperature	High-temperature weather
			Low-temperature weather
		Ventilation Rescue station distance	Indoor ventilation failure
			Outdoor windy weather
			Outdoor wind direction
			Untimely rescue
		Safety facilities	Failure of safety facilities
			Lack of safety facilities
	Management factors (management)	Construction project audit permit situation	Illegal construction/production/operation
			Lack of qualification of outsourcing unit
			Construction project quality is not up to standard
			Defective process design
		System management	Confusion of job responsibilities
			Inadequate supervision and inspection
			Inadequate safety management system
		Process management	Insufficient investigation of hidden dangers
			Missing operating procedures
		Site operation management	Lax operation approval
			Unlicensed/unregulated operations
			Insufficient confirmation of safety conditions before operation
			Wrong assignment of laborers

**Table 3:** Statistics on types and Casualties of large and above chemical accidents from 2000 to 2020.

	Type of Accident	Number of Accidents	Percentage (%)	Number of Casualties	Percentage (%)
Single Type of accident	Leaks (single)	1	0.51	19	0.41
	poisoning choke (single)	35	17.95	345	7.38
	Fire (single)	2	1.03	12	0.26
	Explosion (single)	48	24.62	574	12.28
	Others (Mechanical damage, scald) (single)	2	1.03	24	0.51
Non-single type of accident	leaks, scald	1	0.51	20	0.43
	leaks, poisoning choke	32	16.41	324	6.93
	leaks, fire	6	3.08	48	1.03
	leaks, Explosion	28	14.36	931	19.92
	leaks, fire, Explosion	24	12.31	499	10.68
	fire, poisoning choke	1	0.51	4	0.09
	fire, Explosion	14	7.18	1851	39.61
	fire, Explosion, poisoning choke	1	0.51	22	0.47
Total		195	100	4673	100

**Table 4:** Frequency for causes of large and above accidents each year.

Year	2000	2001	2002	2003	2004	2005	2006	2007	2008	2009	2010
Human	4	2	1	0	4	5	5	10	5	4	6
Machine	2	3	1	1	3	3	2	3	6	4	6
Environmental	0	0	1	1	0	0	0	0	1	0	0
Management	0	0	0	0	3	5	5	4	6	4	3
**Year**	**2011**	**2012**	**2013**	**2014**	**2015**	**2016**	**2017**	**2018**	**2019**	**2020**	**Total**
Human	8	6	11	8	12	11	10	9	13	4	138
Machine	11	6	9	8	11	4	15	8	8	1	115
Environmental	0	0	1	0	2	0	1	3	3	0	13
Management	10	6	12	7	7	8	10	7	11	2	110

**Table 5:** Frequency for causes of large and above accidents each month.

Month	1	2	3	4	5	6	7	8	9	10	11	12	Total
Year	10	12	8	16	9	10	15	13	10	10	13	12	138
Human	10	10	6	12	7	7	15	9	4	8	13	14	115
Environmental	0	1	1	2	1	0	2	2	1	1	2	0	13
Management	6	6	7	17	7	10	13	13	7	9	10	5	110

**Table 6:** Values and ranking of annual systematic risk factor.

Year	$C_{j\;year}$	Rank	Year	$C_{j\;year}$	Rank	Year	$C_{j\;year}$	Rank
2000	0.81817	18	2007	0.57202	9	2014	0.44424	7
2001	0.86740	19	2008	0.60706	11	2015	0.25359	2
2002	0.95442	20	2009	0.72810	15	2016	0.48324	8
2003	0.99895	21	2010	0.62043	12	2017	0.14827	1
2004	0.76979	16	2011	0.30653	5	2018	0.41809	6
2005	0.70581	13	2012	0.57904	10	2019	0.30199	4
2006	0.72449	14	2013	0.28591	3	2020	0.81451	17

**Table 7:** Values and ranking of monthly systematic risk factor.

**Month**	$C_{j\;month}$	**Rank**	Month	$C_{j\;month}$	Rank	Month	$C_{j\;month}$	Rank	Month	$C_{j\;month}$	Rank
1	0.73106	9	4	0.50644	1	7	0.51354	2	10	0.68903	7
2	0.71502	8	5	0.80464	10	8	0.55157	3	11	0.56436	4
3	0.83001	11	6	0.66946	6	9	0.86445	12	12	0.64654	5

## Data Availability

Not applicable.

## Appendix A. LAHCA List in China from 2000 to 2020

**Time**	**LAHCA Details**	**Deaths**	**Injuries**	**Types**
27 January 2000	“1·27” oil pipeline leakage and explosion accident of Guigang Branch of Guangxi Petroleum Company	8	17	leaks, explosion
11 February 2000	The “2·11” explosion accident of individual gas station in Zhangshu, Jiangxi Province	6		explosion
16 February 2000	Explosion accident of “2·16” converting pot in Phosphore Plant of Phosphorus City, Kaiyang, Guizhou	3	2	explosion
2 July 2000	Explosion accident of “7·2” oil tank in Weifang Hongrun Petrochemical Auxiliary Plant, Qingzhou, Shandong	10		leaks, explosion
27 February 2001	“2·27” explosion at Dafeng Fertilizer Plant in Yancheng, Jiangsu Province	5	1	leaks, explosion
23 July 2001	7·23 Explosion at the gas station on Shangcheng Road of Henan Zhengzhou Standard Petrochemical Co., Ltd.	4	12	leaks, explosion
7 November 2001	Leakage and explosion of benzophenone in the “11·7” section of Changfeng Chemical Plant in Changshou County, Chongqing	3	7	leaks, explosion
23 February 2002	Liaoning Liaoyang Petrochemical Olefins hydro carbon Plant “2·23” explosion accident	8	19	leaks, explosion
27 August 2002	“8·27” hydrogen sulfide poisoning accident at Lanzhou Petrochemical Company in Gansu Province	5	45	poisoning choke
17 April 2003	Shandong Liaocheng Lanwei Chemical Co., Ltd. “4·17” poisoning accident	4	133	poisoning choke
16 April 2004	Chongqing Tianyuan Chemical Industry “4·16” nitrogen trichloride explosion chlorine leakage accident	9	3	explosion, leaks
27 October 2004	10·27 explosion at Daqing Petrochemical Plant in Heilongjiang Province	7		leaks, explosion
14 November 2004	“11·14” toxic gas leakage accident of Shandong Jinan Huayang Application Technology Co., Ltd.	3	5	leaks, poisoning choke
13 December 2004	Xishui County, Hubei Province “12·13” explosion of Furuide Chemical Company	3	2	leaks, explosion
26 December 2004	The “12·26” explosion at Chunjiang Company in Changzhou, Jiangsu Province	3		explosion
24 February 2005	Jiangsu Tianyin Chemical Co., Ltd. “2·24” explosion accident	6	11	leaks, explosion
4 April 2005	“4·4” hydrogen sulfide poisoning accident at Sheshan Inorganic Chemical Factory, Songjiang County, Shanghai	4	3	poisoning choke
27 May 2005	Yuncheng County, Shandong County Keda Pharmaceutical Chemical Co., Ltd. “5·27” explosion accident	6	1	explosion
26 July 2005	The 7·26 explosion at Hu Dai Fine Chemical Plant in Wuxi, Jiangsu Province	9	3	leaks, explosion
15 October 2005	“10·15” sulfuric acid leakage accident of Qingdao Oriental Chemical Co., Ltd.	6	13	leaks
13 November 2005	The “11·13” explosion at petrochina Jilihua Biphenyl plant in Jilin Province	8	60	explosion
20 February 2006	Heilongjiang Daqing Oilfield Co., Ltd. “2·20” asphyxiation accident	3		leaks, poisoning choke
29 May 2006	“5·29” fire accident of Lanzhou Petrochemical Company in Gansu Province	4	11	leaks, fire
28 July 2006	Explosion of “7·28” chlorination tower in Linhai Branch of Jiangsu Sheyang Yancheng Fluorine Source Chemical Company	22	29	explosion
4 August 2006	“8·4” poisoning and suffocation accident of Shandong Wucheng Kangda Chemical Co., Ltd.	4	4	poisoning choke,
7 August 2006	Tianjin Yikun Fine Chemical Company “8·7” explosion accident	10	3	explosion
11 December 2006	“12·11” water tank flash accident of Lanzhou Petrochemical Company of petrochina in Gansu	3		explosion
13 January 2007	Jiangsu Kunshan Kangda Pharmaceutical Chemical Company “1·13” reactor explosion accident	7		explosion
26 April 2007	“4·26” explosion in Qingdao Hengyuan Chemical Co., Ltd., Shandong Province	3		explosion
8 May 2007	“5·8” explosion accident of Ganhui Pharmaceutical Chemical Co., Ltd., Xingan County, Jiangxi Province	3	12	explosion
11 May 2007	5·11 explosion accident of Cangzhou Dahua TDI Co., Ltd., China National Chemical Corporation of Hebei	5	80	explosion
11 July 2007	7·11 explosion in Shandong Deqilong Chemical Group Co., Ltd.	9	1	explosion
14 July 2007	Henan Luoyang Runfang Special Oil Co., Ltd. “7·14” poisoning accident	3	1	poisoning choke
27 July 2007	Chongqing Wanzhou Sote salt chemical factory “7·27” poisoning accident	5		poisoning choke
11 October 2007	“10·11” poisoning accident of Shandong Yantai Cashi Industry Co., Ltd.	5		leaks, poisoning choke
24 November 2007	Explosion accident of “11·24” LPG storage tank in Shanghai Pusan Road Oil and Gas Filling Station	4	30	explosion
27 November 2007	Jiangsu Lianhua Technology Co., Ltd. “11·27” deflagration accident	8	5	explosion, fire
28 November 2007	Zhejiang Linghua Industrial Co., Ltd. “11·28” deflagration accident	3		leaks, explosion, fire
23 February 2008	Henan Puyang Zhongyuan Dahua Group Co., Ltd. “2·23” nitrogen asphyxiation accident	3	1	leaks, poisoning choke
12 June 2008	“6·12” hydrogen sulfide poisoning accident of Kunming Anning Qitian Fertilizer Co., Ltd.	6	29	leaks, poisoning choke
2 August 2008	Guizhou Xinghua Chemical Co., Ltd. “8·2” methanol storage tank explosion accident	3	2	leaks, explosion, fire
26 August 2008	8·26 explosion accident of Guangwei Chemical Co., Ltd., Hechi City, Guangxi Zhuang Autonomous Region	21	59	leaks, explosion, fire
14 September 2008	Liaoning Liaoyang Jinhang Petrochemical Co., Ltd. “9·14” explosion accident	3	2	leaks, explosion, fire
17 September 2008	“9·17” chlorine poisoning accident of Yunnan Southern Phosphorus Group Electrochemical Co., Ltd.		71	leaks, poisoning choke
25 November 2008	“11·25” fire accident of Guangdong Luoding Xinbang Forestry Chemical Co., Ltd.	3	3	leaks, explosion, fire
8 December 2008	The “12·8” explosion accident of Huainan Super Strong Chemical Company in Anhui Province	3	2	leaks, explosion
1 January 2009	Explosion accident of acetonitrile unit “1·1” in Shandong HeliKerun Chemical Co., Ltd.	5	9	explosion
12 June 2009	“6·12” hydrogen sulfide poisoning accident of Taizhou Fengrun Biochemistry Company, Zhejiang Province	3	2	poisoning choke
15 July 2009	7·15 explosion accident of Luoran Co., Ltd., Luoyang, Henan Province	8	8	leaks, explosion
10 August 2009	Anhui Fengyuan (Suzhou) Biological Chemical Co., Ltd. “8·10” poisoning death accident	3	1	poisoning choke
3 December 2009	Jiangxi Jiangli Technology Co., Ltd. “12·3” poisoning and suffocation accident	3	2	poisoning choke
14 December 2009	Beijing Guangzhongyuan Gas Company “12·14” deflagration accident	3		explosion, fire
7 January 2010	Petrochina Lanzhou Petrochemical “1·7” tank farm explosion accident	6	6	leaks, explosion
29 June2010	Deflagration accident of “6·29” original oil tank in Liaoning Liaoyang Petrochemical Company	5	5	explosion, fire
4 July 2010	Gansu Baiyin Tianxiang Building Materials Chemical Co., Ltd. “7·4” poisoning accident	3	3	poisoning choke
16 July 2010	7·16 oil pipeline explosion and fire accident of petrochina International Storage and Transportation Co., Ltd. in Dalian, Liaoning Province	2		leaks, explosion, fire
22 July 2010	Guizhou Xingyi Yihua Chemical “7·22” pipeline leakage explosion accident	8	3	leaks, explosion
28 July 2010	“7·28” propylene pipeline leakage and deflagration accident in Nanjing, Jiangsu Province	22	120	leaks, fire, explosion
20 November 2010	Shanxi Yusshe Chemical Co., Ltd. “11·20” explosion accident	4	5	leaks, explosion
2 December 2010	“12·2” explosion accident of Jilantai Chlor-alkali Chemical Company in Alxa League, Inner Mongolia	3	1	explosion
20 December 2010	Gansu Province Xinchuan fertilizer company “12·20” poisoning asphyxiation accident	5	2	poisoning choke
30 December 2010	The “12·30” explosion at Xinxin Biopharmaceutical Company in Kunming, Yunnan Province	5	8	explosion
6 January 2011	Xinjiang Dahuangshan Hongji Coking Co., Ltd. “1·6” gas poisoning accident	3	1	leaks, poisoning choke
18 January 2011	Inner Mongolia Wuhai Chemical Co., Ltd. “1·18” explosion accident	3		leaks, explosion
19 January 2011	Explosion accident of “1·19” heavy oil catalytic unit of Fushun Petrochemical Co., Ltd.	3	4	leaks, explosion
13 March 2011	Luliang County, Yunnan Province Hongying Phosphorus Industry Co., Ltd. “3·13” poisoning and suffocation accident	3	1	leaks, poisoning choke
27 March 2011	Anhui Anqing Xinfu Chemical Co., Ltd. “3·27” explosion accident	3	1	explosion
22 April 2011	Hunan Yanling Huafeng Chemical Co., Ltd. “4·22” explosion accident	6	4	explosion, fire
23 April 2011	“4·23” explosion accident of Nanchong Hongtai Biochemical Co., Ltd., Sichuan Province	4	2	leaks, explosion
28 May 2011	Shandong Zibo Baoyuan Chemical Co., Ltd. “5·28” explosion accident	3	8	explosion, fire
4 August 2011	“8·4” poisoning accident of Ningxia Dawei Teri Pharmaceutical Co., Ltd. in Yongning County, Yinchuan, Ningxia	3	2	poisoning choke
5 August 2011	The “8·5” explosion at Harbin Kaile Chemical Products Factory in Heilongjiang Province	3	1	explosion, fire
13 September 2011	Jiangxi Leping Jiangwei Hi-tech Co., Ltd. “9·13” explosion accident	3	3	explosion
16 October 2011	“10·16” explosion accident of Changshan Insulation Material Co., Ltd., Zhejiang Province	3	3	explosion, fire
6 November 2011	“11·6” explosion and fire accident of Jilin Songyuan Petrochemical Co., Ltd.	4	7	leaks, explosion, fire
19 November 2011	Shandong Xintai United Chemical Co., Ltd. “11·19” deexplosion accident	15	4	leaks, explosion, fire
17 December 2011	Ningxia Baofeng Energy Group Company “12·17” hydrogen sulfide poisoning accident	3	9	leaks, poisoning choke
24 December 2011	Henan Gongyi Wufa auxiliary factory “12·24” explosion accident	3	1	leaks, explosion, fire
4 January 2012	The “1.4” reactor explosion accident of Xiangyang Chemical Plant in Jiaxing, Zhejiang Province	3	4	explosion
4 January 2012	Explosion accident of “1·9” dryer in Scientific Research Institute of Henan Jiaozuo Chemical Plant	4	3	explosion
26 January 2012	Fujian Longyan Zijin copper acid production workshop “1·26” poisoning accident	3	1	poisoning choke
16 February 2012	Gansu Baiyin Lefu Chemical Co., Ltd. “2·16” poisoning accident	3		leaks, poisoning choke
28 February 2012	“2·28” explosion accident of Hebei Zhaoxian Keer Chemical Co., Ltd.	29	46	leaks, explosion
18 April 2012	“4·18” poisoning accident of Anhui Zhongsheng Pharmaceutical Co., Ltd.	3	4	leaks, poisoning choke
15 May 2012	Inner Mongolia Hulunbuir Jinxin Chemical Co., Ltd. “5·15” choking accident	3	2	poisoning choke
25 August 2012	Shandong Guojin Chemical Plant “8·25” explosion accident	3	7	explosion
20 November 2012	Ningxia Zhongwei Xingertai Chemical Co., Ltd. “11·20” poisoning accident	4	2	poisoning choke
1 March 2013	Explosion accident of “3·1” sulfuric acid storage tank of Liaoning Jianping Hongshen Trading Co., Ltd.	7	2	leaks, explosion
29 March 2013	Hebei Wei County Hongshun Chemical Raw Materials Co., Ltd. “3·29” poisoning and suffocation accident	3	2	leaks, poisoning choke
25 April 2013	Liaoning ChemChina Shenyang Paraffin Chemical Co., Ltd. “4·25” hydrogen sulfide poisoning accident	3		poisoning choke
2 June 2013	Large explosion and fire accident of “6·2” in triphenyl tank farm of petrochina Dalian Petrochemical Company in Liaoning Province	4		leaks, explosion, fire
21 July 2013	Gansu Jinshi Chemical Co., Ltd. “7·21” poisoning accident	4	4	poisoning choke
7 August 2013	Zhejiang Ningbo Jiangning Chemical Co., Ltd. “8·7” poisoning and suffocation accident	3		poisoning choke
14 September 2013	Liaoning Fushun Shunte Chemical Co., Ltd. “9·14” explosion and fire accident	5		leaks, explosion, fire
3 October 2013	10·3 Poisoning accident at Hongyanwan Chemical Plant in Baokang County, Hubei Province	3	5	leaks, poisoning choke
8 October 2013	“10·8” major explosion accident of Baoxing County Chengli Gas Supply Co., Ltd.	10	33	leaks, explosion
18 October 2013	“10·18” poisoning accident of Shandong Gugrao County Runheng Chemical Co., Ltd.	3		leaks, poisoning choke
21 October 2013	Shandong Kenli Xinfa Pharmaceutical Co., Ltd. “10·21” fire accident	4	1	leaks, fire
22 November 2013	The “11·22” Sinopec Donghuang oil pipeline leak and explosion in Qingdao, Shandong Province was a particularly serious accident	62	136	leaks, explosion
7 December 2013	The “12·7” liquefied natural gas leak in Yulin, Shaanxi Province	4		leaks, poisoning choke
29 December 2013	The “12·29” explosion at Jiuzhou Chemical Plant in Lanshan District, Linyi City, Shandong Province	3		explosion
1 January 2014	“1·1” naphtha poisoning accident of Shandong Binhua Binyang Burning Chemical Co., Ltd.	4	3	leaks, poisoning choke
9 January 2014	Anhui Kangda Chemical Co., Ltd. “1·9” poisoning accident	4	2	poisoning choke
18 January 2014	Jilin Tonghua Chemical Co., Ltd. “1·18” explosion accident	3	5	explosion
1 March 2014	Sichuan Tianyi Chemical Co., Ltd. “3·1” explosion accident	3		leaks, explosion
8 April 2014	4·8 explosion in Wuhai City, Inner Mongolia Taihe Coal Coking Group Co., Ltd.	3	2	explosion
16 April 2014	4·16 explosion accident of Shuangma Chemical Co., Ltd., Rugao City, Jiangsu Province	9	8	explosion
24 April 2014	Liaoning Lighthouse North Chemical Co., Ltd. “4·24” poisoning and suffocation accident	3		poisoning choke
26 April 2014	Shanxi Yongxin Coal Coking Co., Ltd. “4·26” explosion accident	4	31	leaks, explosion
2 May 2014	Sichuan Guangyuan Tiansen Coal Chemical Company “5·2” explosion accident	3		explosion
29 May 2014	“5·29” explosion at Shuguang Auxiliary Factory in Baoying County, Jiangsu Province	3	3	explosion
1 July 2014	Explosion accident of “7·1” methylamine storage tank of Ningxia Ruitai Technology Co., Ltd.	4	1	explosion
7 July 2014	Deflagration accident of “7·7” chlorobenzene recovery tower of Zhongyi Chemical Industry in Qujing, Yunnan Province	3	4	leaks, explosion, fire
31 July 2014	“7·31” pipeline leakage explosion accident of Taiwan Kaohsiung Hua Yun Storage Company	30	302	leaks, explosion
7 September 2014	Ningxia Jiemei Fengyou Chemical Co., Ltd. “9·7” poisoning accident		41	leaks, poisoning choke
23 September 2014	Hunan Luxiang Barium “9·23” maintenance Ramon machine deflagration accident	6		explosion, fire
8 February 2015	Shandong Guanxian Xinrui Industrial Co., Ltd. “2·8” flash accident	3	5	explosion
19 February 2015	Hubei Zhijiang Fusheng Chemical Co., Ltd. “2·19” explosion accident	5	2	fire, explosion
3 March 2015	Inner Mongolia Tianrun Fertilizer Company “3·3” large scalding accident	3		scald
6 April 2015	Fujian Zhangzhou Tenglong Aromatics (Zhangzhou) Co., Ltd. “4·6” explosion and fire accident		6	leaks, explosion, fire
9 April 2015	Shandong Weifang Binhai Xiangquan Chemical Co., Ltd. “4·9” poisoning and suffocation accident	3	2	poisoning choke
16 May 2015	Shanxi Jincheng Yangcheng Ruixing Chemical Co., Ltd. “5·16” poisoning accident	8	6	leaks, poisoning choke
18 June 2015	“6·18” poisoning accident of Hao-hua Branch of Heilongjiang Beidahuang Agricultural Company	3		poisoning choke
28 June 2015	The “6·28” explosion accident of Ordos Yidong Jiuding Chemical Company in Inner Mongolia	3	6	leaks, explosion
16 July 2015	“7·16” explosion accident of Shandong Shida Petrochemical Co., Ltd., Rizhao City, Shandong Province		2	leaks, explosion, fire
26 July 2015	“7·26” atmospheric pressure unit leakage and fire accident of petrochina Qingyang Petrochemical Company in Gansu Province	3	4	leaks, fire
12 August 2015	Tianjin Port “8·12” Ruihai Company dangerous goods warehouse especially serious fire and explosion accident	173	798	fire, explosion
31 August 2015	“8·31” explosion accident of Shandong Dongying Binyuan Chemical Co., Ltd.	13		explosion
13 October 2015	Xishui County, Hubei Province “10·13” suffocation accident of Xishui Union Gas Co., Ltd.	3		poisoning choke
19 October 2015	Jiangsu Thorp Chemical Construction Engineering Co., Ltd. “10·19” poisoning and suffocation accident	3		poisoning choke
27 November 2015	Heilongjiang Shengnong Science and Technology Development Co., Ltd. “11·27” poisoning accident	3		leaks, poisoning choke
28 November 2015	Hebei Handan Longgang Chemical Co., Ltd. “11·28” liquid ammonia leakage accident	3	4	leaks, poisoning choke
9 January 2016	Shandong Weifang Changxing Chemical Co., Ltd. “1·9” hydrogen fluoride leakage poisoning accident	3	1	leaks, poisoning choke
16 March 2016	Sichuan Jinlu Resin Co., Ltd. “3·16” vinyl chloride poisoning accident	3	2	poisoning choke
1 April 2016	Hebei Handan Daming County Futai Biotechnology Co., Ltd. “4·1” hydrogen sulfide poisoning accident	3	3	leaks, poisoning choke
9 April 2016	Hebei Chengde Xinglong County Tianlihai Flavor & Fragrance Co., Ltd. “4·9” fire accident	4	3	leaks, fire
22 April 2016	Jiangsu Deqiao Storage Co., Ltd. “4·22” large fire accident	1		fire
25 April 2016	Jiangxi Zhangjiang Chemical Co., Ltd. “4·25” deflagration accident	3	1	explosion, fire
5 June 2016	Shandong Weifang Huahao Agrochemical Co., Ltd. “6·5” major drowning and asphyxiation accident	3		poisoning choke
15 June 2016	June 15 Fire accident at Shijiazhuang Refinery and Chemical Plant in Hebei Province	4		fire, poisoning choke
13 July 2016	Shandong Heze City Yuncheng County illegal chemical plant “7·13” larger poisoning and asphyxiation accident	3		poisoning choke
8 September 2016	An illegal dye intermediate production cell “9·8” explosion accident in Jinzhou, Hebei Province	7		explosion
20 September 2016	“9·20” MDI buffer tank burst accident of Shandong Wanhua Chemical Group Co., Ltd.	4	4	explosion
13 November 2016	Hubei Zhongxiang Dasheng Chemical Co., Ltd. “11·13” choking accident	3		poisoning choke
19 November 2016	Hebei Hengshui Tianrun Chemical Technology Co., Ltd. “11·19” poisoning accident	3	2	leaks, poisoning choke
3 January 2017	Zhejiang Linhai Huabang Pharmaceutical Chemical Company “1·3” explosion accident	3		explosion
12 February 2017	Xinjiang Yihua Chemical Co., Ltd. “2·12” carbide furnace spray accident	2	8	explosion
17 February 2017	“2·17” flash explosion accident in Jiangnan Factory of Jilin Songyuan Petrochemical Co., Ltd.	3		explosion
21 February 2017	Inner Mongolia Alxa League Lixin Chemical Co., Ltd. “2·21” explosion accident	2	4	explosion
22 April 2017	Anhui Anqing Wanhua Oil Co., Ltd. “4·2” deflagration accident	5	3	fire, explosion
13 May 2017	“5·13” chlorine poisoning accident of Hebei Lixing Special Rubber Co., Ltd.	2	25	leaks, poisoning choke
5 June 2017	Shandong Linyi Jinyu Petrochemical Co., Ltd. “6·5” explosion and fire accident	10	9	leaks, explosion, fire
9 June 2017	Zhejiang Shaoxing Linjiang Chemical Co., Ltd. “6·9” deflagration accident	3	1	explosion, fire
27 June 2017	“6·27” explosion accident of Wuhai Huazi Coal Coking Company in Inner Mongolia	3		explosion
22 July 2017	Jiangxi Jiujiang City Zhijiang Chemical Company “7·2” explosion accident	3	3	explosion
10 August 2017	“8·10” fire accident of Hebei Cangzhou Zhongjie Petrochemical Co., Ltd.	2	12	leaks, fire
17 August 2017	Liaoning Dalian Petrochemical “8·17” fire accident			leaks, fire
24 September 2017	Hubei Dajiang Chemical Group Co., Ltd. “9·24” major suffocation accident	3		poisoning choke
11 November 2017	Hubei Zhongxiang Golden Eagle Energy Technology Company “11·11” large poisoning accident	3		poisoning choke
18 November 2017	“11·18” poisoning accident of Western Pacific Petrochemical Co., Ltd. in Dalian, Liaoning Province	3	6	poisoning choke
30 November 2017	“11·30” major mechanical injury accident of Xinjiang petrochina Urumqi Petrochemical Company	5	16	mechanical damage
9 December 2017	Jiangsu Lianyungang Juxin Biological Company “12·9” major explosion	10	1	leaks, explosion
19 December 2017	Shandong Rike Chemical Co., Ltd. “12·19” major fire accident	7	4	fire
24 January 2018	Xinjiang Turpan Hengze Coal Chemical Co., Ltd. “1·27” flash accident	3	1	explosion
3 February 2018	Shandong Linyi Jinshan Chemical Co., Ltd. “2·3” large deflagration accident	5	5	leaks, explosion, fire
1 March 2018	Hebei Tangshan Huayi Industrial Company “3·1” large fire accident	4	1	leaks, explosion, fire
9 April 2018	Shanxi Yunyan New Material Co., Ltd. “4·9” major accident	3		poisoning choke
26 April 2018	Tianjin Bohua Yongli Chemical Company “4·26” contractor poisoning and suffocation accident	3	2	leaks, poisoning choke
12 May 2018	Shanghai Sinopec Shanghai Secco “5·12” flash accident	6		leaks, explosion, fire
18 June 2018	“6·18” explosion accident of Chaigang Xingfa Furfural Co., Ltd., Nongan County, Jilin Province	3	3	explosion
20 June 2018	Liaoning Huludao Shixing Pharmaceutical Company “6·20” poisoning and suffocation accident	3		poisoning choke
12 July 2018	Sichuan Yibin Hengda Technology Co., Ltd. “7·12” major explosion accident	19	12	leaks, explosion, fire
28 November 2018	Zhangjiakou, Hebei Province, China National Chemical Group Shenghua Chemical Company “11·28” major deflagment accident	24	21	leaks, explosion, fire
8 December 2018	Henan Energy and Chemical Group Luoyang Yonglong Chemical Company “12·8” poisoning accident	3	1	leaks, poisoning choke
18 December 2018	Jiangsu Rugao Zhongchang Chemical Co., Ltd. “12·18” poisoning accident	3		leaks, poisoning choke
25 December 2018	Xinjiang Turpan City Tokesun Chemical Co., Ltd. “12·25” flash accident	7	14	explosion
3 March 2019	“3·3” hydrogen sulfide poisoning accident in WengFudazhou Chemical Co., Ltd., Sichuan Province	3	3	leaks, poisoning choke
21 March 2019	Jiangsu Xiangshui Tianjiayi Chemical Co., Ltd. “3·21” especially serious explosion accident	78	716	fire, explosion
15 April 2019	Jinan Qilu Tianhe Huishi Pharmaceutical Co., Ltd. “4·15” serious fire poisoning accident	10	12	fire, explosion, poisoning choke
24 April 2019	Inner Mongolia Yidong Group Dongxing Chemical Co., Ltd. “4·24” deflagration accident	4	36	leaks, explosion, fire
22 May 2019	Shaanxi Hengyuan Investment Group Electrochemical Co., Ltd. “5·2” large scalding accident	5	15	leaks, scald
26 June 2019	Henan Kaifeng Xumei Biotechnology Co., Ltd. “6·26” large explosion accident	7	4	leaks, fire, explosion
19 July 2019	“7·19” major explosion at Henan Gas Group Yima Gasification Plant in Sanmenxia City, Henan Province	15	16	leaks, explosion, fire
22 July 2019	Hebei Zhangjiakou City Huailai County Great Wall Biochemical Engineering Co., Ltd. “7·22” major poisoning and asphyxiation accident	5	4	poisoning choke
29 August 2019	Ningxia Zhongwei United Xinli Chemical Co., Ltd. “8·29” explosion accident	4	3	explosion
31 August 2019	Fujian Jian ‘ou Jinfeng Chemical Gas Co., Ltd. “8·31” explosion accident	3		explosion
11 October 2019	Shaanxi Hengxiang Biological Chemical Co., Ltd. “10·11” poisoning and suffocation accident	6		poisoning choke
15 October 2019	Liaoning Chaoyang Jinyao Chemical Products Co., Ltd. “10·15” poisoning accident	3	2	leaks, poisoning choke
15 October 2019	Guangxi Yulin Lanke New Material Technology Co., Ltd. “10·15” explosion accident	4	8	explosion
31 December 2019	Jiangsu Xuzhou Tian ‘an Chemical Co., Ltd. “12·31” poisoning accident	3		leaks, poisoning choke
11 February 2020	Liaoning Huludao Liaoning Xianda Agricultural Science Co., Ltd. “2·11” explosion accident	5	10	explosion
3 August 2020	Hubei Xiantao Blue Silicone Co., Ltd. “8·3” flash accident	6	4	explosion
14 September 2020	Gansu Zhangye Yaobang Chemical Technology Co., Ltd. “9·14” poisoning accident	3		leaks, poisoning choke
14 September 2020	Shanxi Xiaoyi Shanxi Jinmao Energy Technology Co., Ltd. “9·14” poisoning accident	4	1	leaks, poisoning choke
28 September 2020	Hubei Tianmen Chutian Biotechnology Co., Ltd. “9·28” explosion accident	6	1	explosion
